# The efficacy of uterine artery embolization with gelatin sponge for retained products of conception with bleeding and future pregnancy outcomes

**DOI:** 10.1186/s42155-020-00107-4

**Published:** 2020-02-12

**Authors:** Yasushi Kimura, Keigo Osuga, Keisuke Nagai, Hidenari Hongyo, Kaishu Tanaka, Yusuke Ono, Hiroki Higashihara, Shinya Matsuzaki, Masayuki Endo, Tadashi Kimura, Noriyuki Tomiyama

**Affiliations:** 1 Department of Diagnostic and Interventional Radiology, Osaka University Graduate School of Medicine, 2-2 Yamadaoka Suita, Osaka, Japan; 2 Department of Radiology, Osaka Medical College, 2-7 Daigaku-machi Takatsuki, Osaka, Japan; 3 Department of Obstetrics and Gynecology, Osaka University Graduate School of Medicine, 2-2 Yamadaoka Suita, Osaka, Japan

**Keywords:** Retained products of conception, Post-partum hemorrhage, Uterine artery embolization

## Abstract

**Background:**

Retained products of conception (RPOC) with hemorrhage need intervention when RPOC persist and remain symptomatic. The safety and efficacy of uterine artery embolization (UAE) for RPOC using gelatin sponge (GS) alone, and fertility after UAE for RPOC remain unknown. The purpose of this study is to retrospectively investigate the efficacy of UAE for RPOC with bleeding and future pregnancy outcomes.

**Methods:**

Between 2007 and 2016, 14 patients (mean age, 33 years old) diagnosed as RPOC with bleeding received UAE using GS at our institution. Pregnancy outcomes were vaginal delivery (*n* = 7), miscarriage (*n* = 4), and termination (*n* = 3). Four patients received dilation and curettage/evacuation (D&C/E) for treatment of RPOC before bleeding occurred. The mean time interval from the end of pregnancy to bleeding was 28 days. Technical success, clinical success, complications, angiographic features and fertility after UAE were retrospectively assessed.

**Results:**

Technical success was achieved in 13 patients (93%) and clinical success was achieved in all 14 patients. No major complications occurred. The angiographic features of RPOC were tortuous feeders with flow into a focal blush of contrast (*n* = 14). Additional findings were pseudoaneurysm (*n* = 6), early venous return (*n* = 4), and extravasation (*n* = 2). Pseudoaneurysm was observed significantly more often in patients who received D&C/E before UAE compared to those who received conservative treatment alone (*P* = 0.015). The mean follow-up period was 29 months. Six patients achieved six pregnancies an average of 29 months after UAE.

**Conclusion:**

UAE using GS may be an effective and safe treatment for RPOC with hemorrhage that can preserve fertility.

## Background

Retained products of conception (RPOC) is defined as a condition of persistent placental tissue in the uterine cavity after abortion or delivery. The prevalence of RPOC is estimated at approximately 1% of term pregnancies and considered to be more common after miscarriage or termination (Romero et al., 1990). Presence of RPOC induces various symptoms, such as fever, abdominal pain, and abnormal genital bleeding (Hakim-Elahi et al., 1990). As RPOC often disappear spontaneously, treatment is often conservative with careful observation (Jain & Fogata, 2007; Lee et al., 2014). If RPOC persist and remain symptomatic, gynecologic interventional options include dilation and curettage/evacuation (D&C/E), hysteroscopic resection (HR) and hysterectomy (Ben-Ami et al., 2014; Golan et al., 2011; Aseeja, 2012; Porreco & Stettler, 2010). However because 18% of RPOC has marked vascularity (Kamaya et al., 2009), gynecologic interventional options can be invasive and have the possibility of life-threatening hemorrhage (Kamaya et al., 2009; Kitahara et al., 2011). Uterine artery embolization (UAE) for RPOC has been reported as a case report (Kitahara et al., 2011) or case series (Bazeries et al., 2017), but the safety and efficacy of UAE for RPOC using gelatin sponge (GS) alone, and fertility after UAE for RPOC remain unknown.

The purpose of this study is to retrospectively investigate the efficacy of UAE for RPOC with bleeding and future pregnancy outcomes.

## Methods

### Patients

This was a single center, retrospective study, which was approved by the ethics committee of our hospital. Between 2007 and 2016, 14 UAE procedures were performed for RPOC with bleeding in 14 patients. All patients experienced abnormal bleeding after delivery, miscarriage, or termination. The diagnosis of RPOC was performed by an obstetrician considering clinical examination features and findings on imaging such as doppler ultrasound (US, *n* = 14), contrast enhanced computed tomography (CE-CT, *n* = 8) or contrast enhanced magnetic resonance imaging (CE-MRI, *n* = 3). The mean age was 33 (±5) years old. Median gravidity was 2 (range 1–4). Three of the 14 patients conceived after assisted fertility treatments. Pregnancy outcomes were vaginal delivery in seven patients (50%), miscarriage in four patients (29%), and termination in three patients (21%). All patients received conservative treatment including uterine contraction drugs (oxytocin, misoprostol or methylergometrine) and hemostatic agent (carbazochrome and tranexamic acid). The mean hemoglobin level of the 14 patients was 7.7 g/dL (range 5.3–9.9). Eight patients (57%) received blood transfusion (red blood cells and/or fresh frozen plasma). The mean volume of transfusion before UAE was 4.8 units of red blood cells (range, 0–12 unit) and 2.7 units of fresh frozen plasma (range, 0–12 unit). Four patients received D&C/E for treatment of RPOC before bleeding occurred. The mean time interval from the end of pregnancy to bleeding was 28 (±16) days. The US findings before UAE were an endometrial mass with vascularity in 8 patients (57%), an endometrial mass without vascularity in 4 patients (29%), and abnormal vascularity in the endometrium in 2 patients (14%). The patients’ characteristics are shown in Table 1.

**Table 1:** Patients characteristics (*n* = 14)

Age, years, mean (±SD)		33 (±5)
Reproductive history, n (%)	G1	6 (43%)
	G2	3 (21%)
	G3	4 (29%)
	G4	1 (7%)
Fertility treatment, n (%)		3 (21%)
Pregnancy outcomes, n (%)	Vaginal delivery	7 (50%)
	Miscarriage	4 (29%)
	Termination	3 (21%)
Bleeding after D&C/E for RPOC, n (%)		4 (29%)
Hemoglobin, g/dL, mean (range)		7.7 (5.3–9.9)
Transfusion, unit, mean (range)	Red blood cell	4.8 (0–12)
	Fresh frozen plasma	2.7 (0–12)
Bleeding onset after the end of pregnancy, days, mean (±SD)		28 (±16)
Ultrasound findings before UAE	Intrauterine mass with vascularity	8 (57%)
	Intrauterine mass	4 (29%)
	Hyper-vascularity in endometrium	2 (14%)

### Angiography and UAE techniques

Angiography was performed via a unilateral femoral artery approach under local anesthesia. In all cases, a 4 French sheath was inserted, and aortography was performed using a pigtail catheter to check the arterial anatomy of the pelvis and decide the side of UA to embolize first by identifying bleeding point. Aortography may be omitted depending on the situation, such as immediate treatment is preferred. After aortography, selective uterine artery (UA) angiography was performed to confirm and localize RPOC using a Mohri-type (looped cobra-shaped) catheter (4Fr.,. Hanako Medical Co., Ltd., Saitama, Japan) with a microcatheter (Nadeshiko Swanneck 2.4Fr., JMS Co., Ltd., Hiroshima, Japan). Embolization was performed using GS via a microcatheter after selective UA angiography. A GS sheet (Serescue, Nippon Kayaku Co., Ltd. Tokyo, Japan) was sliced into three sheets with a scalpel, then the sliced sheet was squashed to about 1–2 mm thick with scissors body or syringe. The sheet was then cut into 1-2 mm square to make cube particles. These particles were collected in 10 mL syringe, diluted with contrast and saline at the ratio of 1:1, and slowly injected under fluoroscopy. In one case, ready-made porous gelatin particles (Gelpart, Nippon Kayaku, diameter: 1 mm and 2 mm) were used according to the operator’s preference. Embolization was performed beyond the vaginal branch of the uterine artery. The endpoint of embolization was contrast stasis over five cardiac beats under fluoroscopy. Finally, hemostasis was confirmed by bilateral UA angiography and gynecological examination.

### Study endpoints and definitions

Technical success, clinical success, complications, angiographic features, and future pregnancy were retrospectively assessed. Technical success was defined as embolization of the bilateral uterine arteries or unilateral uterine artery of the responsible side with contrast stasis over five cardiac beats under fluoroscopy. Clinical success was defined as no re-bleeding during the follow-up period. Major and minor complications were evaluated according to the criteria of the Society of Interventional Radiology (Drooz et al., 1997). After UAE, patients were followed up by outpatient visits with obstetricians at our institution, typically twice a month, until no abnormality in uterus was confirmed. The obstetric charts were collected and reviewed to gather data about US findings, menstruation recovery, clinical signs and symptoms, and outcomes of subsequent pregnancies.

### Statistical analysis

The characteristics of the angiographic findings in patients with conservative treatment were compared with those in patients with post D&C/E bleeding using the Fisher’s exact test. All statistical analyses were performed by using GraphPad Prism ver. 8.00 (GraphPad Software, San Diego California USA).

## Results

### Technical success and clinical success

Technical success rate of UAE with gelatin sponge for RPOC with bleeding was 93% (13 patients). Technical failures was observed in one patient with a arteriovenous shun between the right UA and the right internal iliac vein. The left UA was a source of bleeding from RPOC and embolized with success. Right UA embolization was tried in case the shunt caused bleeding. Embolization with small amount of GS was performed, but no angiographical change was observed after embolization. Additional embolization was avoided because possible passage of the hand-cut GS to venous system was concerned. After procedure, hemostasis was confirmed by the obstetrician. Unilateral UAE was performed in 4 cases including one technical failure case. The responsible side was evident in all four cases and embolized successfully. Clinical success was obtained in all 14 patients, including technical failure case and unilateral UA embolization. All patients remained free from re-bleeding after the mean follow-up period (29 months, range: 1–127 months).

### Complications

Major complications were not observed during or after UAE. As minor complications, post-embolization syndromes such as lower abdominal pain occurred in 8 cases (62%), fever in 5 cases (38%), nausea in 3 cases (23%), and vomiting in 2 cases (15%). These symptoms resolved with conservative management such as nonsteroidal anti-inflammatory drugs for postembolization fever.

### Angiographic findings

**Fig. 1:**
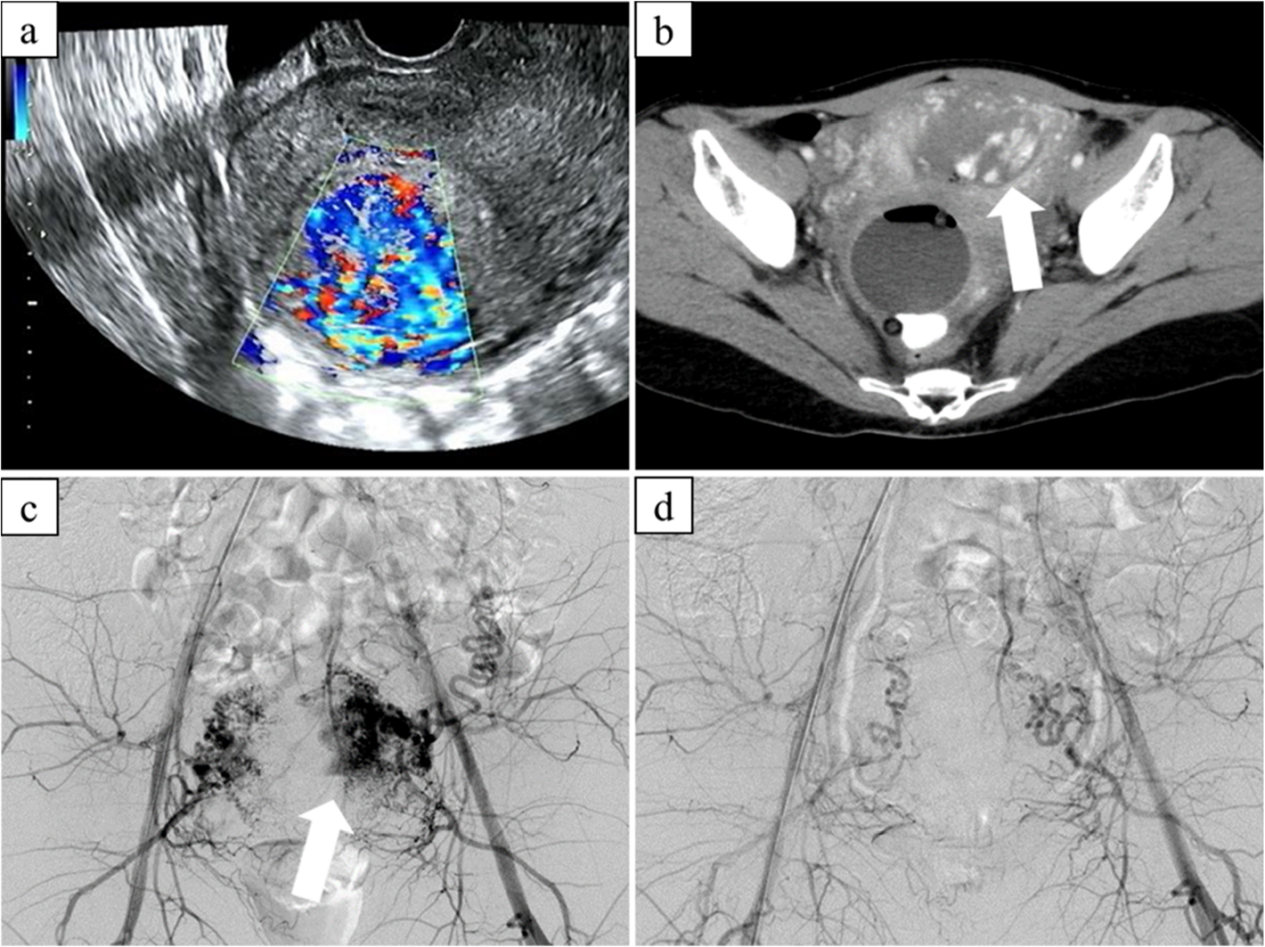
31-year-old patient with vaginal bleeding after vaginal delivery. **a** Transvaginal ultrasonography showing a hypervascular hyperechoic mass in the endometrium. **b** Arterial-phase contrast enhanced CT showing focal enhancement (arrow) within the endometrium. **c** Aortography showing numerous spiral arteries and a focal blush in the left side of uterus (arrow). **d** Post-embolization aortography showing contrast stasis without focal blush

**Fig. 2:**
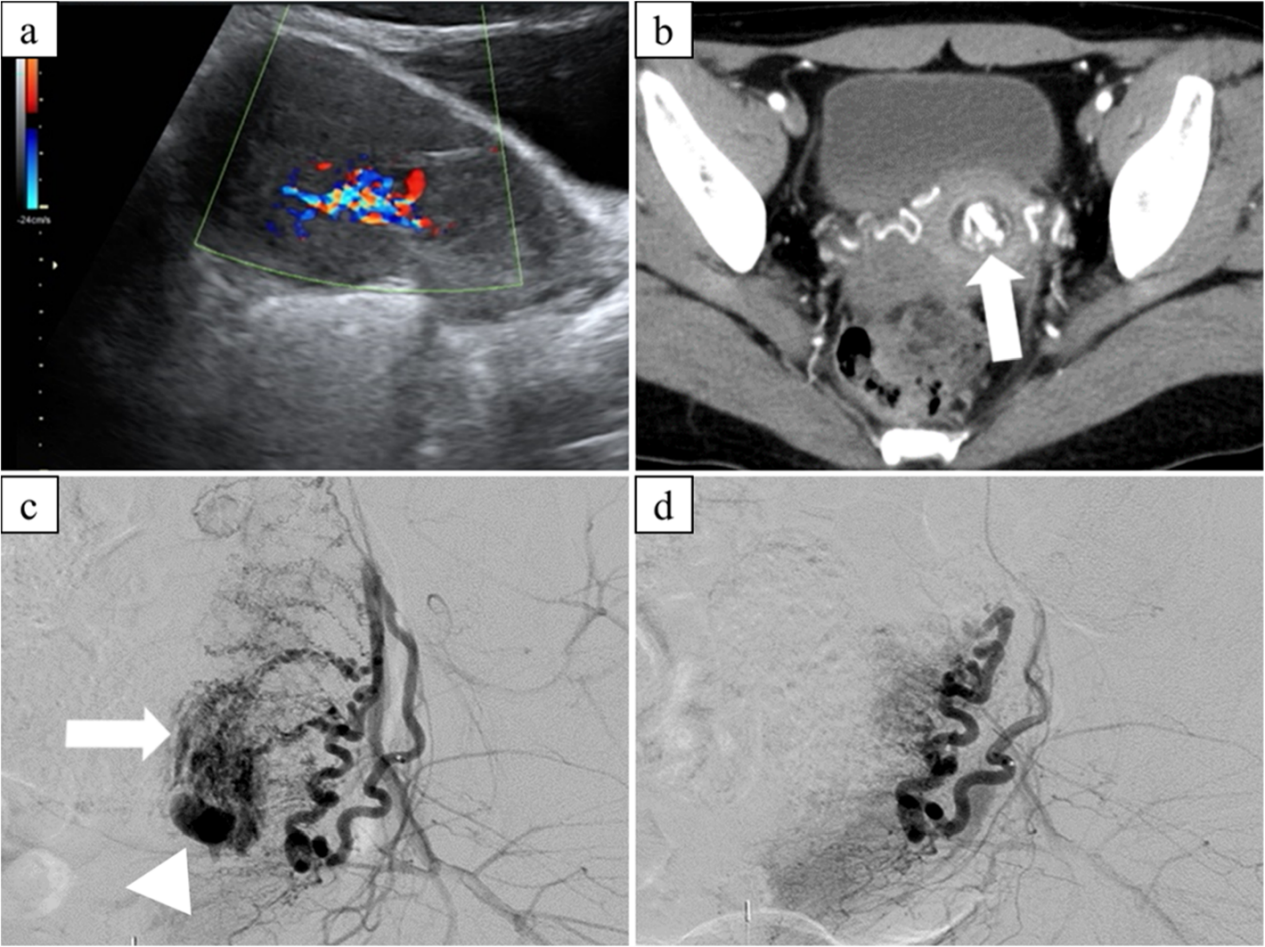
32-year-old patient with vaginal bleeding post D&C for missed abortion. **a** Trans-abdominal ultrasonography showing a hypervascular intrauterine mass. **b** Arterial-phase CE-CT showing an enhancing lesion in the uterine cavity (arrow). **c** Left uterine arteriogram showing a focal blush (arrow) and a pseudoaneurysm (arrowhead). **d** Post-embolization left uterine arteriogram confirming the disappearance of the pseudoaneurysm and focal blush

Angiographic findings are summarized in Table 2. On UA angiography, a focal blush of contrast was observed in all cases (Fig. 1). There was no significant difference in the degree of depiction of the focal blush, early venous return or extravasation. Pseudoaneurysm (Fig. 2) was observed significantly more often in patients who received D&C/E before UAE compared to those who received conservative treatment alone (*P* = 0.015). In two patients, bleeding was seen from extra-uterine arteries (the vaginal branch of left inferior vesical artery and the left ovarian artery in one patient each). The left ovarian artery was not embolized to prevent ovarian ischemia.

**Table 2:** Angiographic findings

		Conservative treatment alone (*n* = 10)	Post D&C/E (*n* = 4)	*P* value*
Focal blush of contrast	Obvious Faint	7 (70%) 3 (30%)	3 (75%) 1 (25%)	.5
Early venous return		4 (40%)	0 (0%)	.25
Pseudoaneurysm		2 (20%)	4 (100%)	.015
Extravasation		1 (10%)	1 (25%)	.5

*Fisher’s exact test

### Follow-up and pregnancy outcomes

Follow-up US findings were loss of blood flow, thinning of the endometrium and/or disappearance of remnants. The mean follow-up period was 29 months (range, 1–127 months). All patients remained free from re-bleeding and symptoms caused by RPOC during follow-up. Three patients ended follow-up in 1 months and ongoing observation by local physicians. Nine out of the rest 11 patients resumed menstruation after UAE and seven of the nine tried to conceive. At the end of follow-up, six patients achieved six pregnancies an average of 29 months after UAE (range, 15–60 months). Among them, one patient achieved pregnancy 3 years after UAE with fertility treatment. All were full term pregnancies with uneventful delivery and all babies were healthy.

## Discussion

In this study, RPOC with bleeding were treated by UAE using GS with high technical and clinical success rates. In one technical failure case, we couldn’t embolize right UA because of a direct shunt between the right UA and the right internal iliac vein. However clinical success was obtained; the bleeding source from RPOC was considered as left UA and embolized successfully. The arteriovenous shunt itself might not be a source of bleeding because uterine arteriovenous shunt can be asymptomatic and result in spontaneous lesion resolution (Degani et al., 2009).

In recent case series of UAE for RPOC using mainly microspheres (size: 700-1200 μm) (Bazeries et al., 2017), 25.8% of patients required additional treatment for re-bleeding. Microspheres may be unsuitable for bleeding from RPOC with markedly dilated spiral arteries in the inter-villous space of the placental tissue (Roberts, 2014), which may be up to 2 mm in diameter (Espinoza et al., 2006). We experienced ovarian supply in one case, which was managed without embolization. The ovarian artery supplied only small part of RPOC on angiography and both uterine artery embolization was enough to stop bleeding caused by RPOC. However, ovarian arteries might be one of the causes of re-bleeding: ovarian artery supply was confirmed in 12% of PPH patients (Kim et al., 2018). Therefore, it is suggested that we should visualize both ovarian artery on initial aortography at the level of the renal arteries and selective embolization might be needed if its contribution to bleeding is confirmed (Kim et al., 2018; Maassen et al., 2009). Pseudoaneurysm was more frequently seen on angiography in patients after D&C/E in this study. This suggests that the traumatic procedure of D&C/E can cause pseudoaneurysm due to arterial rupture of a hypervascular RPOC. Thus, vascularity should be evaluated with color Doppler before D&C/E for RPOC (Kitahara et al., 2011). Also, dynamic gadolinium-enhanced MRI may support the US findings regarding the vascularity of the mass to avoid bleeding complication related to gynecologic interventions (Dohke et al., 2000).

A standard of care for RPOC is usually careful observation because RPOC often disappear spontaneously without treatment (Jain & Fogata, 2007; Lee et al., 2014). When RPOC remains symptomatic, gynecologic options such as D&C/E and HR (Ben-Ami et al., 2014; Golan et al., 2011) can be one of options. However, it might have the possibility of life-threatening hemorrhage because 18% of RPOC have marked vascularity on doppler US (Kamaya et al., 2009). There is no reliable means to predict future spontaneous massive bleeding (Kitahara et al., 2011), therefore conservative management may be preferable to emergency UAE, if the patient’s condition allows. As not-embolization methods to manage RPOC, ‘maximum laminaria procedure’ with surgical RPOC removal was recently reported by Usui et al. (Usui et al., 2018). They performed overnight insertion of two laminaria tents into cervical canal of patients with expect of the compression of uterine artery. This might be one of choices to treat RPOC with non-urgent bleeding, but UAE might be preferred for cases of massive bleeding.

Uterine arteriovenous malformation (AVM) is a differential diagnosis of RPOC, although rare with 0.1% of after abortion or delivery to 4.5% of patients with abnormal vaginal bleeding (Yazawa et al., 2013; O’Brien et al., 2006). The torturous abnormal feeding arteries and early venous filling seen in RPOC in this study are similar angiographic findings to those of uterine AVM (Timmerman et al., 2003; Ghai et al., 2003). To discriminate congenital uterine AVM from RPOC, US finding of mass-like lesion in the endometrium or thickened endometrial echo complex is a key feature of RPOC (Sellmyer et al., 2013), but strict differentiation may be difficult, because RPOC and AVM can have overlapping and can coexist (Iraha et al., 2018). Management of symptomatic uterine AVM resembles RPOC with bleeding: surgery or UAE. UAE provides a safe and effective alternative to surgery, with 88–100% success rate using coils, GS and n-butyl-2-cyanoacrylate (Badawy et al., 2001; Wang et al., 2012; Picel et al., 2016).

In this study, menstruation after UAE was observed in nine out of 11 patients (82%) whose follow up period was longer than 1 month. Pregnancy was observed in six of the nine (67%) patients with menstruation after UAE. A recent study showed that 92.5% of patients resumed menstruation after UAE (Gaia et al., 2009). The difference in our study might be because these two patients experienced normal delivery although follow-up period was only two and 3 months. After UAE using GS, 62% of patients were reported to achieve pregnancy (Gaia et al., 2009), almost the same rate as UAE using NBCA; 60% pregnancy rate (Picel et al., 2016). UAE might have possibility of pregnancy, but future research should address unknown effect for uterine function and complication in long period of time.

There are some limitations in our study. First, this is a retrospective study with a small number of patients. RPOC with massive bleeding are rare disease, and a multicenter registry may be warranted to assess the embolization techniques and outcomes. Second, histopathological confirmation of RPOC was not undertaken except in one case. In addition, despite RPOC spontaneously detached or diminished after UAE, further examination (hysteroscopy or follow-up CE-MRI) might be needed because remnant tissues degeneration has potentials for future malignancy. Yet it might be addressed that RPOC is one of a main cause of late PPH and may be managed by UAE using GS which might have possibility of fertility preservation.

## Conclusion

For managing bleeding caused by RPOC, UAE using GS may be an effective and safe treatment with a high technical and clinical success rate. UAE with GS might become an important option in this subgroup of patients who wish to retain the option for future pregnancy.

## Data Availability

All data gathered or analyzed in this study are included in this article.

## Abbreviations

D&C/E: Dilation and Curettage/Evacuation
GS: Gelatin Sponge particles
HR: Hysteroscopic Resection
RPOC: Retained Products Of Conception
UAE: Uterine Artery Embolization
